# Orofacial Pain, Musical Performance and Associated Coping Behaviors, Psychological Distress and Disability among Asian Young Adults

**DOI:** 10.3390/jcm12041271

**Published:** 2023-02-06

**Authors:** Rahul Nair, Chihiro Tanikawa, Joao N. Ferreira

**Affiliations:** 1 Department of Dentistry—Quality and Safety of Oral Healthcare, Radboud University Medical Centre, Radboud Institute for Health Sciences, 6525 Nijmegen, The Netherlands; 2 Faculty of Dentistry, National University of Singapore, Singapore 119085, Singapore; 3 Department of Orthodontics and Dentofacial Orthopedics, Graduate School of Dentistry, Osaka University, Osaka 565-0871, Japan; 4 Avatar Biotechnologies for Oral Health and Healthy Longevity Research Unit, Faculty of Dentistry, Chulalongkorn University, Bangkok 10330, Thailand

**Keywords:** musculoskeletal pain, orofacial pain, temporomandibular disorders, mental disorders, coping behaviors, disability, music

## Abstract

Musicians often report orofacial pain (OFP) and performance-related psychological distress related to occupational neuromuscular overuse, but to date, no study has been performed in Asian musicians to assess these factors. This study evaluated OFP, psychological distress, coping behaviors, and disability among Asian musical performers. A total of 201 participants in Singaporean music ensembles were surveyed from which 159 met the inclusion criteria for vocalists or instrumentalist musicians (mean age 20.26 ± 2.20 years). Self-administered questionnaires assessed musical practices, jaw/neck pre-conditioning exercises, pain-related temporomandibular disorders (TMD), OFP descriptors, pain chronicity and disability, coping behaviors and psychological distress. Univariate and multi-variate analyses were carried out. OFP, while performing, was more than two-fold higher in instrumentalists when compared to vocalists (41.4–48% vs. 17.2%, *p* = 0.002). A similar trend occurred for OFP that progresses while playing (*p* = 0.035) and for persistent OFP that reduces playing (*p* = 0.001). There were no differences in psychological distress, pain coping and disability between groups. Vocalists were found to practice jaw/neck pre-conditioning exercises more frequently (75% vs. 4–12.9% in instrumentalists, *p* < 0.0001). While performing, Asian vocalists reported less OFP when compared to instrumentalists. Future prospective studies are needed to confirm if pre-conditioning exercises play a protective role against OFP in vocalists.

## 1. Introduction

Orofacial musculoskeletal pain conditions can often arise from the temporomandibular joint (TMJ) and its associated musculoskeletal structures [1,2,3,4]. These painful conditions as well as headaches and neck/shoulder pains can develop after musculoskeletal overuse of the masticatory and cervical muscles due to persistent and repetitive oro-mandibular activities, oral behaviors, or postural habits [3,4,5]. Certain professions such as musicians may be more predisposed to this repetitive orofacial muscular strain related to their musical practices and performance [5,6,7,8]. Two case–control studies suggested a possible association between temporomandibular disorders (TMD) and playing violin/viola [6,7]. More recent questionnaires performed in Dutch ensembles indicated that TMD pain was associated with playing woodwind instruments, and violin or viola players reported pain in the neck and shoulder [5]. More importantly, oral behaviors seem to stand as a risk indicator.

It is thought that playing musical instruments that are persistently held between the mandible and the shoulder, could overload and burden the orofacial musculoskeletal system, thereby possibly causing pain [6,9]. Indeed, this musculoskeletal overload may also occur with wind and brass instruments (e.g., clarinet, oboe, trumpet) as they involve the creation of an embouchure that is sustained by the facial muscles in coordination with the masticatory muscles, which can lead to orbicularis oris focal dystonias and recurrent myalgias [9]. Vocal performers or vocalists earlier appeared to report high levels of orofacial musculoskeletal pain potentially due to persistent mouth opening activities [10], but more recent literature [11] comparing them with other musicians indicates singing is not a risk factor for TMD pain. Systematic reviews on this particular topic are not possible due to lack of studies with moderate-to-high methodological quality [12].

The existing large orchestra surveys and systematic review studies had assessed prevalence of orofacial pain (OFP) and other bodily pains in professional musicians mainly in Europe and Australia [5,11,13,14,15]. Pain prevalence among musicians varies widely in these countries but can go well above 50% [5,13]. Frequency of oral behaviors appears to be associated with TMD-related pain and temporomandibular joint sounds [11]. Stage fright has been reported to be a predictor for persistent playing-related musculoskeletal pain [15]. Hence, resilience coping behaviors and psychological factors may be key factors to preventing pain-related disability, sick leave, and premature career termination [15,16]. In the Asian, highly-competitive musical performance context, young adult musicians at early career stages may face important challenges in terms of OFP coping and psychological distress. There is currently a lack of OFP studies in Asian musicians to determine if such factors are risk indicators.

Thus, the objective of this study was to assess OFP, musical performance, psychological distress, and pain-related disability in young Asian adult musicians with different musical practices. We hypothesize that specific groups of musicians and musical practices may be more prone to OFP, poor pain coping behaviors, and high levels of psychological distress and pain-related disability. An evaluation of jaw/neck pre-conditioning exercises (before musical practice) was included as a secondary outcome.

## 2. Materials and Methods

### 2.1. Participants

Self-administered paper-based questionnaires were used for data collection among young Singaporean adults from 3 music ensembles (chamber music ensembles, symphony orchestras, fanfares, choirs) at the Yong Siew Toh Conservatory of Music, Center for the Arts and Faculties at the National University of Singapore (NUS) campus from October 2014 until July 2016. The questionnaires were delivered by our research team to make sure they were properly filled before each musical rehearsal. The protocol for this study survey was approved by the NUS IRB Ethical Committee (reference code: B-14-205E) and an informed consent from each participant was obtained according to the Declaration of Helsinki. Musicians filled the questionnaires after rehearsal, a procedure that took less than 10 min. Participants were considered “active” musicians if they were currently playing a musical instrument (named instrumentalists) or were vocalists (abbreviated as “Vocal”). An expert panel composed of two members of our research team with extensive musical expertise (J.N.F., C.T.) and two faculty musicians from the NUS Music Conservatory determined by mutual agreement the instruments that typically require active jaw tasks that could burden orofacial structures such as the jaw and muscles. Based on this panel determination, instrumentalists were further categorized into 2 groups based on whether they required or not oromandibular activities to play their instruments: (1) “Active Jaw” and (2) “Non-active” Jaw (Table 1).

### 2.2. Survey Questionnaires

A general information questionnaire collected the demographic data, and the following questions were added to assess musical practice confounders according to previous systematic studies and surveys [12,13,15,16]: 1. instrument(s) most often used/played?; 2. total number of years of practice?; 3. average number of daily continuous practice minutes?; 4. maximum number of daily continuous practice minutes?; 5. how often do you take breaks after playing?; 6. how often do you perform jaw and/or neck exercises to warm up your muscles before you start performing?; 7. have you ever had head, face, jaw, and/or neck injuries while performing?.

The overall survey also included these 6 self-assessment questionnaires (SAQs) because they have been previously tested for validity and reliability in young adults with pain and/or psychological distress [17,18,19,20,21,22]: Temporomandibular Disorders Pain Screener (TMD-PS) for assessing OFP/TMD pain, Graded Chronic Pain Scale version 2.0 (GCPS) for determining characteristic pain intensity (CPI) and OFP chronicity, duration and pain-related disability days, Brief Resilient Coping Scale (BRCS) for evaluating resilient coping behaviors, Generalized Anxiety Disorder 7-item scale (GAD-7) to assess level of anxiety severity, Patient Health Questionnaire 4-item scale (PHQ-4) for evaluating psychological distress, and Perceived Stress Scale (PSS) to measure stress perception.

Outcome variables from musical performance-related pains were assessed with questions formulated according to previous surveys [12,13,15,16]. These questions were: in the last 30 days, did you have any of the following pains: a. OFP while playing or for a short period after playing?; b. OFP that progresses while playing and requires the practice session to be shortened and does not resolve between sessions?; c. OFP that progresses while playing and does not requires the practice session to be shortened and resolves between sessions?; d. OFP that persists for a longer period (hours) after playing? All questionnaires were tested on 10 musician participants to check for face and content validity and understanding. This type of validity testing evaluated all aspects of the OFP construct that it is designed to measure and on whether it measures what is supposed to. These participants were told of the intent of the study and were interviewed to assess any cultural adaptation that may be needed for the questionnaires and the instructions of such. Necessary changes in the filling instructions were made, but the selected questionnaires did not raise any need for modifications.

### 2.3. Statistical Analysis

The demographic data, musical practice-related variables (the prevalence of playing-related OFP, TMD pain, OFP descriptors, pain chronicity and disability, coping, anxiety severity, psychological distress and perceived stress) were described as numbers and percentages, and were stratified to the three musician categories.

Univariate analyses were performed first to study the associations between OFP symptoms (while playing) and musical practice parameters (years of musical experience, hours of musical practice per day, muscular pre-conditioning exercises before practice) by using chi-square for categorical orofacial outcomes, or Kruskal–Wallis tests for numerical outcomes since most of the variables were not normally distributed. Bonferroni correction was applied for all comparisons. Finally, multi-variate analyses, using either logistic regression for binary outcomes or linear regression for numerical outcomes, was run to confirm such associations, taking into consideration all confounders (identified in the univariate analysis).

The level of statistical significance for all tests was set at 5%. All data were analyzed using SPSS™ version 24 (IBM Corp., Armonk, NY, USA).

## 3. Results

A total of 201 music ensemble participants consented and took the survey with a completion rate of 95%. Out of these, 159 musicians were found by our expert panel to meet the inclusion criteria for currently active vocalists (*n* = 64) and instrumentalist musicians (*n* = 95). Out of the 95 instrumentalists, 70 played an instrument that requires persistent oromandibular activities, hence were allocated to the “Active Jaw” group, and 25 played an instrument that does not require such jaw activities and were allocated to the “Non-active Jaw” group.

The mean age of all the musicians was 20.26 ± 2.20 years. As for gender, 65 (40.9%) of musicians were males and 94 (59.1%) were females. The majority of musicians were Chinese (*n* = 146, 91.8%). Table 2 shows the mean age and frequencies for gender and race for the three musician groups (Vocal, Active Jaw, Non-active Jaw). There were no statistical differences with regard to age, gender, and race among the three groups. In the analyses of all musical practice related variables, both the musical experience, duration of musical practice, and the frequency of jaw/neck muscular pre-conditioning exercises (before musical practice) were significantly different among the three musician groups (*p* = 0.015, *p* = 0.010, *p* < 0.0001, respectively) as shown in Table 2. Vocalists had less musical experience (26.6% had ≥10 years, vs. 42.9% for Active Jaw and 36% Non-active Jaw groups), but often performed muscular pre-conditioning exercises (75%, vs. 12.9% for Active Jaw and 4% for Non-active Jaw groups). The presence of musical practice breaks and history of orofacial trauma were not statistically different between groups (Table 2).

In terms of painful TMD, musicians requiring oromandibular activities to play instruments together with vocalists who had the highest prevalence of self-reported TMD pain (11.4% and 12.5%, respectively), but these did not statistically differ from the other musician group not requiring such activities (4%, *p* = 0.681), as shown in Table 3.

Among the three groups, there were no statistical differences in OFP descriptors or characteristics such as average CPI, pain chronicity, and pain-related disability. With regard to pain chronicity, the median OFP duration was considerably low across groups (ranging from 0 to 2 days), which indicates the overall acute nature of pain symptoms among these surveyed young adults, although vocalists and “active jaw” instrumentalists reported chronic pain lasting 90 days or more. Overall, these self-reported pain characteristics may imply that OFP had negligible impact on pain-related disability days in all musician groups (median of zero in all groups).

Next, we investigated differences between pain associated behavioral and psychosocial co-variates among musicians (Table 4).

There were no statistical differences between the three musician groups relative to coping behaviors, anxiety, psychological distress, and perceived stress. The average median scores ranging 14–14.5 across musician groups indicated medium resilient coping behaviors; however, the range of scores fell below 14 and above 16, which also points out to a wide scope of coping behaviors. As for anxiety, all median scores (range 5–5.5) indicate a mild anxiety severity level in the three groups. Likewise, median scores ranging from 3 to 4 in the PHQ-4 scale also suggest mild psychological distress in all groups. As for perceived stress, median scores were at moderate level (range 17–18).

Thus, the next step was to understand how OFP symptoms fluctuate with the musical performances and distinctive practices of each musician group. For this purpose, we used face validity questionnaires adopted by previous surveys [12,13,15,16]. Interestingly, while exploring the presence of OFP while performing or playing music (Table 5), we found that it was significantly higher (more than two-fold) in the two instrumentalist groups when compared to vocalists (41.4–48% vs. 17.2%, *p* = 0.002).

This was also the case for OFP that progresses while playing (30–36% vs. 14.1%, *p* = 0.035), and persistent OFP that reduces playing (12–20% vs. 0%, *p* = 0.001). Multivariate logistic regression was then conducted, as displayed in Table 6, for these two pain outcomes that appear to occur during musical practice: OFP while playing and OFP that progresses after playing.

The following variables, musician groups (Vocal, Active Jaw, Non-active Jaw), musical experience (1–3 years, 4–9 years, and >9 years), duration of each musical practice (<30 min, 30 min–60 min, and >60 min), and jaw/neck muscular pre-conditioning exercise (once a day/everyday, never) were included in the model. Due to statistical limitations, logistic regression could not be run for the outcome “OFP that reduces playing” since it was 0% for the vocalist group. The findings are summarized in Table 6, where one can observe that instrumentalists are significantly more likely to have OFP while playing and shortly afterwards when compared to vocalists (for Active Jaw group: OR = 3.819, *p* = 0.015; for Non-active Jaw group: OR = 4.214, *p* = 0.028) when adjusted to all other variables. Moreover, the difference for having “OFP that progresses while playing” between instrumentalists and vocalists was close to the boundary of significance (for Active Jaw group: OR = 2.904, *p* = 0.085; for Non-active Jaw group: OR = 3.646, *p* = 0.069), and most of the confidence intervals were disproportionately over 1. As for variables such as musical experience, duration of each musical practice and jaw/neck muscular pre-conditioning exercises, the associations with “OFP while playing” and “OFP that progresses while playing” were not significant.

## 4. Discussion

### 4.1. OFP Prevalence and Musical Practice Outcomes

The main goal of this study was to evaluate OFP, musical performance, psychological distress and pain-related disability in Asian young adult musicians with different musical practices. In this singular pain study among Asian musicians, the prevalence of TMD-related pain was not significantly different between instrumentalists and vocalists while measured by TMD-PS (Table 3). However, during musical performance, the frequency of OFP while playing in vocalists was twice less than instrumentalists (17.2% and 43.2%, respectively, Table 5). In a survey including 306 Dutch vocal performers, self-reported temporomandibular disorder (TMD)-related pain among vocalists was 21.9%, vs. 12.0% in other musicians [11]. In contrast, in the study herein, OFP associated with TMD was comparable between vocalists and instrumentalists requiring oro-mandibular activities (12.5% and 11.4%, respectively). However, in the same Dutch study, being a vocalist was neither a risk indicator for TMD pain nor for TMJ sounds [11]. In fact, the self-reported TMD pain among musicians was positively correlated with female gender, frequency of oral behaviors, and hours of daily practice. These lower self-reported playing-related pain symptoms suggest that vocalists may benefit from their regular vocal training including jaw/neck muscular pre-conditioning exercises, which was significantly more common in our surveyed vocalist group (75%). Moreover, the years of musical experience and the duration of daily practice sessions may potentially place instrumentalists and even vocal performers at risk for developing OFP while playing, similar to the study in Dutch musicians [11], though these did not increase the odds for OFP symptoms in our multivariate regression model. Low levels of TMD-related pain symptoms have been reported in a sample study with 50 Brazilian vocalists, though their main focus was on body pain, and no gold standard TMD questionnaires were used for assessing pain [23].

### 4.2. OFP Chronicity, Disability, and Psychosocial Factors among Musicians

Furthermore, in our survey, TMD-related pain frequency rates had minimal impact on both OFP chronicity and number of pain-related disability days in all musician groups. Similar findings were reported in a large cross-sectional study to assess TMD symptoms in instrumentalist musicians. In a German survey, up to 10% of 408 instrumentalists from orchestras reported painful TMD or craniomandibular disorders [8]. Similarly, in the United States, a national survey on musculoskeletal symptoms/disorders assessed among 2212 musicians also found comparable TMD prevalence rates (11%), although the survey questionnaires did not objectively ask about pain in the orofacial areas [24]. In Australia, a survey on 377 orchestra musicians also reported similar outcomes for TMD [14]. These prevalence rates for TMD pain are comparable to the general population according to epidemiological studies performed in North America [1,2,4]. A common limitation of these large musician surveys is their focus on collecting data solely on musculoskeletal pain symptoms, not including potential confounders such as pain chronicity, psychological distress, and pain coping behaviors, which are important to control stage fright [16]. In our survey done on pain in Asians, there were no major differences in coping behaviors, anxiety severity, psychological distress levels, perceived stress, pain chronicity, and disability among musicians (vocalists and instrumentalists). It is important to note the fact that this study was conducted before the current coronavirus pandemic period; hence, the prevalence of these psychosocial co-variates may have changed with the several public mandatory lockdowns and stay home government policies driven by the pandemic.

In the Asian context, no OFP surveys have been reported targeting specifically musicians. A study conducted in the general population in Hong Kong reported that approximately 30% of 1526 Cantonese-speaking individuals reported jaw pain via a telephone interview, particularly females [25]. Yap and others [26] have found higher TMD pain prevalence and psychological distress levels in Singaporean women in community dental clinics. In our study on musicians, there were no gender differences for orofacial and TMD pains (data not shown). In musicians, large cross-sectional studies done in Germany and the Netherlands have indicated that female gender, stage fright, frequency of oral behaviors, and duration of musical practice are associated with musculoskeletal pains [11,15].

### 4.3. Limitations of the Study

This study had a limited sample size of musicians with chronic OFP to allow for multifactorial analysis and comparisons in terms of psychosocial factors, perhaps because the sampled population only included young adults at early career stages. At the start of this study (2014), a sample size calculation could not be performed due to lack of OFP/TMD prevalence studies in musicians. Matched non-musician controls could have increased the efficiency of the study, but they would have dwindled the statistical power. Other potential limitation could be the lack of control of other variables that could influence the SAQs and the measures they report. Body self-awareness and questionnaire fatigue for example can influence the final scoring of each questionnaire. In addition, our sampled music ensembles were composed of musicians with mild levels of OFP intensity and disability, probably because these individuals have high degree of musculoskeletal synaptic plasticity and resilience due to their young age [3]. When comparing to other studies [5,11,16], the main strength of this study was that it uniquely assessed potential psychosocial confounders for pain in musicians, such as pain coping behaviors, and psychological distress, as well as pain-related disability. None of the psychosocial confounders were found to be related with any specific musician group.

### 4.4. Future Research

This study can be further expanded to include professional musicians that practice for longer hours on average (well above 60 min/session). Nevertheless, our study outcomes can inform dental professionals and other relevant healthcare practitioners in the prevention and management of OFP (e.g., TMD pain) in instrumentalists to evade disability throughout their future professional career stages. Moreover, it is important for the dental professional to seek measures that can prevent or alleviate the predisposition of instrumentalists towards orofacial and neck musculoskeletal pains, including TMD. One approach has successfully used oral splints to manage TMD in musicians; however, randomized clinical trials (RCTs) are still required to evaluate the efficacy of these and other conservative TMD therapies [27]. Such therapies may also include jaw/neck muscular pre-conditioning exercises as well as jaw/body postural awareness and relaxation techniques, such as the Alexander technique (AT), to prevent or manage further musculoskeletal pains or oro-mandibular injuries (e.g., dystonias) [28,29]. Evidence from RCTs and clinical trials indicate that AT sessions may improve stage fright or performance-related anxiety in musicians [29]. Cervical stabilization exercise programs seem to be beneficial for musicians playing specific instruments, for example the violin, particularly if non-specific neck pain symptoms are present [30]. At early stages, musicians may also have widespread pains below neck level, affecting shoulders, hands, and wrists [31]. However, effects on music performance, respiratory function, sleep, and jaw/neck posture are yet to be investigated in musicians.

## 5. Conclusions

Taken together, this was a distinctive study performed in Asian music ensembles assessing OFP occurrence during and after playing, TMD pain, musical practices, pain-related disability, coping behaviors, and psychological distress levels. The main findings indicate that vocalists have less OFP symptoms while performing when compared to instrumentalists; nonetheless, pain co-variates such as coping, psychological distress, and disability are similar among musicians. There was no difference between vocalists and instrumentalists specifically regarding painful TMD. Jaw/neck muscular pre-conditioning and relaxation techniques before musical performance possibly play a protective role against orofacial pain in vocal performers, but this requires future prospective studies.

## Figures and Tables

**Table 1:** Definition criteria for all musicians and their allocation into the 3 musician categories according to expert panel.

Musician Categories	Definition Criteria	Musical Instruments
Vocal	Vocalists	Not applicable
Active Jaw	Musicians that require oromandibular activities to play an instrument.	Violin, Viola, Clarinet, Trombone, Euphonium, French Horn, Trumpet, Oboe, Tuba, Flute, Bassoon, Harmonica, Saxophone
Non-active Jaw	Musicians that do not require oromandibular activities to play an instrument.	Piano, Guitar, Cello, Guzheng, Harp, Drum, Keyboard

**Table 2:** Comparison of demographic and musical practice related co-variates among the 3 different musician groups.

	Vocal	Instrumentalists		*p* Value
		Active Jaw	Non-Active Jaw	
	(*n* = 64)	(*n* = 70)	(*n* = 25)	
Age (y), mean ± SD	20.67 ± 1.6	20.20 ± 1.4	19.40 ± 4.2	0.068
Gender, *n* (%)				0.568
Male	27 (42.2)	30 (42.9)	8 (32)	
Female	37 (57.8)	40 (57.1)	17 (68)	
Race, *n* (%)				0.056
Chinese	61 (95.3)	62 (88.6)	23 (92)	
Non-Chinese	3 (4.7)	8 (11.4)	2 (8)	
Musical experience (years), *n* (%)				0.015
1–3	19 (29.7)	5 (7.1)	3 (12)	
4–6	18 (28.1)	19 (27.1)	5 (20)	
7–9	10 (15.6)	16 (22.9)	8 (32)	
≥10	17 (26.6)	30 (42.9)	9 (36)	
Average duration of each musical practice (min), *n* (%)				0.01
<30	37 (57.8)	23 (32.9)	14 (56)	
30–60	20 (31.3)	33 (47.1)	11 (44)	
>60	7 (10.9)	14 (20)	0 (0)	
Breaks during musical practice, *n* (%)				0.344
No	11 (17.2)	7 (10)	2 (8)	
Yes	53 (82.8)	63 (90)	23 (92)	
History of trauma while playing, *n* (%)				0.75
No	59 (92.2)	63 (90)	24 (96)	
Yes	5 (7.8)	7 (10)	1 (4)	
Jaw/neck pre-conditioning exercises, *n* (%)				<0.0001
No	16 (25)	61 (87.1)	24 (96)	
Yes	48 (75)	9 (12.9)	1 (4)	

**Table 3:** Comparison of pain outcomes such as TMD pain prevalence, characteristic pain intensity (CPI), pain chronicity, pain-related disability among musicians.

	Vocal	Active Jaw	Non-Active Jaw	*p* Value
	(*n* = 64)	(*n* = 70)	(*n* = 25)	
TMD pain (TMD-PS), *n* (%)				
No	56 (87.5)	62 (88.6)	24 (96)	0.681
Yes	8 (12.5)	8 (11.4)	1 (4)	
CPI (0–10)	0.15 (0–7)	0 (0–6)	0 (0–4)	0.756
Median (range)				
Pain duration/chronicity, days	2 (0–90)	0 (0–180)	0 (0–20)	0.193
Median (range)				
Pain-related disability days	0 (0–7)	0 (0–5)	0 (0–2)	0.805
Median (range)				

**Table 4:** Comparison of psychosocial factors and co-variates among musicians.

	Vocal	Active Jaw	Non-Active	*p* Value
			Jaw	
	(*n* = 64)	(*n* = 70)	(*n* = 25)	
Resilient Coping Behaviors	14 (10–20)	14.5 (8–20)	14 (9–18)	0.475
Median (range)				
Anxiety severity	5.5 (0–17)	5 (0–19)	5 (0–11)	0.443
Median (range)				
Psychological distress	3 (0–10)	3.5 (0–9)	4 (0–7)	0.89
Median (range)				
Perceived stress	17 (7–28)	17 (5–27)	18 (7–26)	0.722
Median (range)				

**Table 5:** Prevalence of different performance-related orofacial pains (OFP) among musicians.

Types of Performance Related OFP	Vocal	Active Jaw	Non-Active	*p* Value
			Jaw	
	(*n* = 64)	(*n* = 70)	(*n* = 25)	
OFP while playing, *n* (%)				0.002
No	53 (82.8)	41 (58.6)	13 (52)	
Yes	11 (17.2)	29 (41.4)	12 (48)	
OFP that progresses while playing, *n* (%)				0.035
No	55 (85.9)	49 (70)	16 (64)	
Yes	9 (14.1)	21 (30)	9 (36)	
OFP that persists after playing, *n* (%)				0.113
No	55 (85.9)	52 (74.3)	17 (68)	
Yes	9 (14.1)	18 (25.7)	8 (32)	
OFP that reduces playing, *n* (%)				0.001
No	64 (100)	56 (80)	22 (88)	
Yes	0 (0)	14 (20)	3 (12)	

**Table 6:** Association of the musician groups, musical experience, musical practice duration, and muscular pre-conditioning exercises with the presence of performance-related orofacial pains (OFP) as described by odds ratio (OR) and confidence intervals (CI).

		Dependent Variables					
		OFP while Playing			OFP That Progresses while Playing		
		OR	95% CI	*p* Value	OR	95% CI	*p* Value
Musician groups	Active Jaw vs. Vocal	3.819	1.298–11.236	0.015	2.904	0.862–9.786	0.085
	Non-active Jaw vs. Vocal	4.214	1.173–15.143	0.028	3.646	0.906–14.676	0.069
Musical Experience	4–6 vs. 1–3 years	0.988	0.338–2.890	0.982	0.325	0.105–1.010	0.052
	≥10 vs. 7–9 years	1.077	0.350–3.316	0.897	0.749	0.243–2.309	0.614
Duration of Each Musical Practice	30–60 min vs. <30 min	0.545	0.247–1.204	0.133	0.697	0.296–1.640	0.408
	>60 min vs. <30 min	0.929	0.308–2.798	0.896	0.537	0.144–2.007	0.355
Jaw/Neck	Once a day/Everyday vs. Never	0.946	0.341–2.627	0.915	0.746	0.235–2.372	0.62
Pre-conditioning Exercises							

## Data Availability

Data is available upon reasonable request to authors.
